# Necrology

**Published:** 1897-11

**Authors:** 


### NECROLOGY.

DR. EDWARD LOOMES, of New York City, died early in October of acute gastric trouble, and his remains were laid at rest in Woodlawn Cemetery. Dr. Loomes was a highly respected practitioner as well as a very successful one. He was a charter member of the New York County Veterinary Medical Association. He had been appointed veterinarian for the National Horse Show of America, which position he had successfully filled in the past.

